# Using Digital Measurement–Based Care to Address Symptoms of Inattention, Hyperactivity, and Opposition in Youth: Retrospective Analysis of Bend Health

**DOI:** 10.2196/46578

**Published:** 2023-04-26

**Authors:** Darian Lawrence-Sidebottom, Landry Goodgame Huffman, Jennifer Huberty, Clare Beatty, Monika Roots, Kurt Roots, Amit Parikh, Rachael Guerra, Jaclyn Weiser

**Affiliations:** 1 Bend Health, Inc. Madison, WI United States; 2 FitMinded, Inc. LLC Phoenix, AZ United States

**Keywords:** digital mental health intervention, attention-deficit/hyperactivity disorder, opposition defiance disorder, attention deficit, collaborative care, behavioral care, mental health, adolescent, child, hyperactivity, hyperactive, inattention, ADHD, use, caregiver, behavioral problem

## Abstract

**Background:**

Attention-deficit/hyperactivity disorder (ADHD) and associated behavioral disorders are highly prevalent in children and adolescents, yet many of them do not receive the care they need. Digital mental health interventions (DMHIs) may address this need by providing accessible and high-quality care. Given the necessity for high levels of caregiver and primary care practitioner involvement in addressing ADHD symptoms and behavioral problems, collaborative care interventions that adopt a whole-family approach may be particularly well suited to reduce symptoms of inattention, hyperactivity, and opposition in children and adolescents.

**Objective:**

The purpose of this study is to use member (ie, child and adolescent) data from Bend Health, Inc, a collaborative care DMHI that uses a whole-family approach to address child and adolescent mental health concerns, to (1) determine the effects of a collaborative care DMHI on inattention, hyperactivity, and oppositional symptoms in children and adolescents and (2) assess whether the effects of a collaborative care DMHI vary across ADHD subtypes and demographic factors.

**Methods:**

Caregivers of children and adolescents with elevated symptoms of inattention, hyperactivity, or opposition assessed their children’s symptom severity approximately every 30 days while participating in Bend Health, Inc. Data from 107 children and adolescents aged 6-17 years who exhibited clinically elevated symptoms at baseline were used to assess symptom severity across monthly assessments (inattention symptom group: n=91, 85.0%; hyperactivity symptom group: n=48, 44.9%; oppositional symptom group: n=70, 65.4%). The majority of the sample exhibited elevated symptoms of at least 2 symptom types at baseline (n=67, 62.6%).

**Results:**

Members received care for up to 5.52 months and attended between 0 and 10 coaching, therapy, or psychiatry sessions through Bend Health, Inc. For those with at least 2 assessments, 71.0% (n=22) showed improvements in inattention symptoms, 60.0% (n=9) showed improvements in hyperactivity symptoms, and 60.0% (n=12) showed improvements in oppositional symptoms. When considering group-level change over time, symptom severity decreased over the course of treatment with Bend Health, Inc, for inattention (average decrease=3.51 points, *P*=.001) and hyperactivity (average decrease=3.07 points, *P*=.049) but not for oppositional symptoms (average decrease=0.70 points, *P*=.26). There was a main effect of the duration of care on symptom severity (*P*<.001) such that each additional month of care was associated with lower symptom scores.

**Conclusions:**

This study offers promising early evidence that collaborative care DHMIs may facilitate improvements in ADHD symptoms among children and adolescents, addressing the growing need for accessible and high-quality care for behavioral health problems in the United States. However, additional follow-up studies bolstered by larger samples and control groups are necessary to further establish the robustness of these findings.

## Introduction

Attention-deficit/hyperactivity disorder (ADHD) affects approximately 10% of children and adolescents in the United States [1] and is characterized by persistent symptoms of inattention, hyperactivity, or both, resulting in impairment in multiple settings (eg, home and school) [2]. Although it is most often diagnosed and treated in childhood, ADHD can persist past adolescence and may continue to cause impairments into adulthood [3]. Oppositional defiant disorder (ODD) is highly comorbid with ADHD; indeed, a large body of research suggests that ADHD and ODD share a common etiology characterized by disrupted executive function and inhibitory processing [4]. As such, those with ODD often exhibit similar symptoms to ADHD (eg, inattention) accompanied by additional emotional and behavioral problems related to antisociality [5]. An estimated 50% of children with ADHD have a comorbid behavior or conduct problem, such as ODD [6].

The current recommendations for ADHD management involve both behavioral therapy and medication [7]. Nonetheless, only about a third of children and adolescents with ADHD receive behavioral therapy combined with medication for treatment, whereas over half regularly take medication and less than half receive behavioral treatment [7]. ADHD medication is associated with several adverse side effects, and pharmacological treatment may not adequately address nonspecific impairments (eg, poor social skills and low academic achievement) [8]. Further, difficulty adhering to pharmacological treatment plans may be exacerbated by the executive dysfunctions associated with ADHD, which is a particular barrier to treatment in children [9]. Various nonpharmacological interventions can address the symptoms of ADHD [10], including cognitive behavioral therapy (CBT), neurofeedback, physical exercise, cognitive training, and dietary modifications [11-14]. Additionally, interventions delivered via caregivers, including modifications to parenting techniques or targeted support from schoolteachers, may be particularly effective for school-aged children [15].

Given that ADHD is an externalizing disorder involving age-inappropriate behaviors, the Centers for Disease Control and Prevention (CDC) recommends training for parents of those with ADHD or related behavior disorders, especially younger children [16]. Indeed, research suggests that involvement from caregivers is a salient predictor of symptom progression [17]. Behavioral training aims to give caregivers the skills to reinforce and encourage appropriate behaviors, while discouraging inappropriate behaviors. Parent training has been associated with increases in positive parenting and decreases in child ADHD symptom severity [18,19]. An estimated 3 in 10 children and adolescents with ADHD receive care via parent training, and an estimated 6 in 10 receive support from school [7].

To adequately address symptoms and nonspecific impairments, many people with ADHD must leverage several types of care (eg, medication, therapy, and caregiver training). As such, collaborative care models, in which primary care providers (PCPs) partner with other behavioral health professionals (eg, coaches, therapists, psychiatrists), may be especially advantageous for those with ADHD and other complex mental and behavioral problems. Collaborative care models often incorporate measurement-based care, or the regular evaluation of symptoms to facilitate continual optimization of individualized care plans [20]. Indeed, collaborative care improves mental health treatment outcomes, especially for those with depression and anxiety [20-23]. Although clinicians agree that the best practice in ADHD treatment should use collaborative care [24,25], ADHD-focused assessments of collaborative care efficacy are limited.

Digital health platforms have emerged to meet the growing demand for accessible mental health care [26,27]. Given that an estimated 23% of children and adolescents with ADHD are not currently receiving any pharmacological or nonpharmacological treatment [7], digital mental health interventions (DMHIs) may address this need for high-quality ADHD care. DMHIs may be particularly well suited to deliver care to traditionally underserved populations (eg, ethnic minorities and those living in rural areas [25]) and also younger people, as children and adolescents are likely to have easy access to the internet and an existing familiarity with online platforms [28-30].

Although there is evidence supporting the use of DMHIs to address mental and behavioral health problems in children and adolescents [31,32], few target ADHD symptoms using whole-family and collaborative care approaches. Online gamelike therapeutic devices and other digital activities have been developed to treat symptoms of ADHD, especially inattention [33-36]. ADHD is 1 of the most common diagnoses of children and adolescents referred to telepsychiatry by their PCP [37]. However, findings are mixed regarding whether teletherapy benefits those with ADHD [38,39]. There are also current investigations of the use of DMHIs to deliver training to parents of children with ADHD, but these findings are not yet available [39].

Given the high need for caregiver and PCP involvement in ADHD behavioral care, we would expect that collaborative care DMHIs may be particularly effective for those with ADHD. However, to the best of our knowledge, no studies have investigated the effectiveness of collaborative care DMHIs that take a whole-family approach to address elevated symptoms of ADHD among youth. Therefore, the purpose of this study was to use member (ie, children and adolescent) data from Bend Health, Inc, a collaborative care DMHI that uses a whole-family approach to address child and adolescent mental health concerns, to (1) determine the effects of a collaborative care DMHI on inattention, hyperactivity, and oppositional symptoms in children and adolescents and (2) assess whether collaborative care DMHI effectiveness may be modulated by various factors, including symptom type and demographics.

## Methods

### Design and Participants

Children and adolescents (aged 6-17 years) receiving treatment from Bend Health, Inc, a collaborative care DMHI, between May and December 2022 were eligible for inclusion in the study. Scores on the inattention, hyperactivity, and oppositional dimensions of the 26-item Swanson, Nolan, and Pelham (SNAP-IV) ADHD validated measure were used to determine whether a member was included in the retrospective analysis. Specifically, members with baseline scores indicating at least mildly severe inattention, hyperactivity, or oppositional symptoms were included in the analyses of that symptom subset. Scoring and cutoff scores were determined using previously validated SNAP-IV scoring norms.

### Treatment

The treatment with Bend Health, Inc, has been described previously [31]. In brief, Bend Health, Inc, is a collaborative care DMHI for children and adolescents. Bend Health, Inc, uses a whole-person, whole-family treatment approach, encouraging regular involvement from parents, guardians, and other caregivers. After a member is enrolled and assessed, a behavioral care coordinator (BCC), coordinates with the member’s PCP as well as other relevant care team members (eg, psychiatrist, therapist, or coach) to determine the member’s care program (eg, the plan of care developed based on a member’s presenting symptoms and age). The BCC then oversees the execution of the member’s care program under the direction of the PCP. Each member’s care plan partners the child or adolescent’s mental health care team with the child’s PCP and includes synchronous video-based (virtual) care sessions and asynchronous instant messaging between the member’s caregiver and the BCC. Members and caregivers also have access to informational resources via the online platform. Every 30 days, the caregiver is required to complete online assessments of mental health outcomes, including inattention, hyperactivity, and oppositional symptoms.

Bend Health, Inc, coaching sessions are intended to provide the member and their families with appropriate evidence-based behavior change tools, help members with self-reflection, strengthen self-efficacy and autonomy, and, when appropriate, serve as a gateway to additional mental health support via sessions with a licensed therapist. Bend Health, Inc, therapy sessions are intended to provide diagnostic clarity to inform a clinical framework, uncover potential sources of unwanted and targeted behaviors, and address trauma or other complicated clinical psychopathology. Coaching and therapy sessions are based on CBT, behavioral activation, parent management training, mindfulness-based cognitive therapy, motivational interviewing, and mindfulness-based stress reduction. All coaches and therapists are trained in these modalities. Bend Health, Inc, psychiatric providers contribute to the member’s initial and working diagnoses and provide ongoing treatment recommendations to the PCP regarding medication management, psychotherapy services, and any emergent needs for higher levels of care. Members are assigned a care coordinator, along with a single or multiple care providers (which may include a coach, a therapist, or a psychiatrist), based on their initial assessment and treatment needs as well as their pathway to care (eg, PCP referral, insurance benefits, employer benefits, or direct to consumer). All members who enroll at Bend Health, Inc, through a PCP referral meet with a psychiatrist as part of their care plan, which is standard practice within the collaborative care model.

All care programs are adapted to be developmentally appropriate for those receiving care from Bend Health, Inc, with modifications in the care program based on member age. For example, programs for children (members aged 12 years or younger) require an adult caregiver to be present in synchronous sessions, during which the caregiver actively engages with and assists their child throughout the program. Programs for adolescents (members aged 13-17 years) do not require caregivers to attend sessions with their child. However, adolescent programs still include aspects that involve and support the caregiver during and between sessions, and caregivers are required to be readily accessible throughout sessions (eg, in the same general area). Program components, such as scripts and tasks, are also adapted to match cognitive and emotional abilities across a range of ages, such that a child-oriented program includes simpler language and tasks (eg, drawing versus writing) and an adolescent-oriented program includes more complex language and tasks.

### Ethical Considerations

All Bend Health, Inc, members above the age of 12 years (adolescent members and participating caregivers) complete informed consent prior to enrolling in services. Caregivers consented on behalf of their children aged 12 years and under. The informed consent process included essential information about Bend Health, Inc, telemedicine services and privacy policies. Given this study was a retrospective analysis, it was classified as exempt from consent requirements under human subject review and approved by an independent BRANY Institutional Review Board (IRB; study ID 23-12-034-1374, January 16, 2023). Study data were deidentified and stored on a Health Insurance Portability and Accountability Act (HIPAA)–compliant online drive using industry-standard encryption. Participants received no additional compensation for participation.

### Study Measures

Upon enrollment, caregivers were asked to complete demographic information and screening questions to assess for potential elevated inattention, hyperactivity, and oppositional symptoms. These screening questions were intended to flag members with elevated inattention, hyperactivity, or oppositional symptoms, while minimizing the workload for caregivers of members who do not present these symptoms. Participating caregivers were asked, *During the past 2 weeks, how much (or how often) has your child*, and then they selected the best-fit response to each item using a 5-item Likert scale (0=not at all to 4=nearly every day). For inattention and hyperactivity, the screening question was, *Had problems paying attention when they were in class or doing their homework or reading a book or playing a game*. For oppositional symptoms, the screening question was, *Had problematic behaviors in relation to others.* If a caregiver’s response to the inattention and hyperactivity screening question was greater than or equal to 1 (*rare, less than a day or two*), they were prompted to complete questions 1-18 of the SNAP-IV validated measure. If their response to the oppositional/defiance screening question was greater than or equal to 1 (*rare, less than a day or two*), the caregiver was required to complete questions 19-26 of the SNAP-IV validated measure.

The SNAP-IV measure was developed for caregivers to report on the behaviors and symptoms of children and adolescents aged 6-18 years [40], and SNAP-IV scores show good concordance with clinical evaluations of ADHD [41]. The assessment consists of 26 questions in total, with questions 1-9 assessing inattention symptoms, questions 10-18 assessing hyperactivity symptoms, and questions 19-26 assessing oppositional symptoms. Caregivers were instructed to respond to each statement on behalf of their child using a 4-item Likert scale (0=not at all [like my child] to 4=very much [like my child]). Assessment scores were reported to the caregiver on the member portal and used to guide the patient’s care plan.

### Statistical Analysis

The total scores for each subset in the SNAP-IV measure (ie, inattention, hyperactivity, and oppositional) were calculated by adding the values of the individual responses. For the inattention and hyperactivity subsets, scores less than 13 indicated that the symptoms were not clinically significant, scores between 13 and 17 indicated mild symptom severity, scores between 18 and 22 indicated moderate symptom severity, and scores 23 or greater indicated severe symptoms. For the oppositional subset, scores less than 8 indicated that the symptoms were not clinically significant, scores between 8 and 13 indicated mild symptom severity, scores between 14 and 18 indicated moderate symptom severity, and scores 19 or greater indicated severe symptom severity.

Each member’s delta score was calculated as *final subset score (last assessment) – baseline subset score (first assessment)* to quantify the change in the subset score from baseline to the end of treatment. As such, negative change scores indicated an improvement (decrease) in symptom severity. One-tailed Wilcoxon signed-rank tests were conducted to determine whether change scores for inattention, hyperactivity, or oppositional symptoms were significantly less than 0.

The average response to each item (possible range=0-3) was calculated for each subset for further analysis of the change in symptom severity over time using linear mixed effects modeling. The basic model included the fixed effects of months from baseline (time) and symptom type (inattention, hyperactivity, and oppositional) and their interaction, with a random effect of subject (member identification) on the intercept. Additional potential predictors were added to the model 1 at a time as fixed effects, and then the fit of each alternative model was compared to the basic model using likelihood ratio tests. If the potential predictor improved model fit (ie, *P*<.05), it was retained in the final model. The following potential predictors were assessed for each model: average number of Bend Health, Inc, sessions (with a coach, therapist, or psychiatrist) per month, age at baseline, and sex at birth (male or female). The number of months from baseline was used as the time variable because there was variability in the duration between assessments. For example, one member’s second assessment could take place 25 days after baseline, while another’s could take place 40 days after baseline. The primary linear mixed effects model was repeated on data from the first 3 assessments for only those with at least 3 total assessments to assess the robustness of our findings to differences in care duration and associated attrition of available data.

For all analyses, group trends were reported with standard descriptive statistics, including percentage of sample (%), mean (SD), and median (IQR). Due to low response rates to assessments past the fourth for all subsets, descriptive statistics are not reported past assessment 3. Data were analyzed with RStudio version 2023.03.0+386 [42,43].

## Results

### Participant Details

A total of 107 members (aged 6-17 years at enrollment) met the inclusion criteria for at least 1 of the symptom groups. Specifically, at baseline, 91 (85.0%) members had elevated inattention symptoms, 48 (44.9%) members had elevated hyperactivity symptoms, and 70 (65.4%) members had elevated oppositional symptoms. Most members had elevated symptoms on multiple subsets at baseline (Table 1). Due to differences in the duration of participation with the collaborative care DMHI, the rates of SNAP-IV measure completion decreased over assessments (Table 2).

**Table 1:** Rates of elevated symptoms for each subset at baseline.

Subset of elevated symptoms		Participants (N=107), n (%)
**One subset with elevated symptoms**		40 (37.4)
	Inattention only	26 (65.0)
	Hyperactivity only	1 (2.5)
	Oppositional only	13 (32.5)
**Two subsets with elevated symptoms**		32 (29.9)
	Inattention and hyperactivity	10 (31.3)
	Inattention and oppositional	20 (62.5)
	Hyperactivity and oppositional	2 (6.3%)
Three subsets with elevated symptoms (inattention, hyperactivity, and oppositional)		35 (32.7)

**Table 2:** Rates of assessment completion for each subset group.

Assessment	Inattention (n=91), n (%)	Hyperactivity (n=48), n (%)	Oppositional (n=70), n (%)
1 (baseline)	91 (100.0)	48 (100.0)	70 (100.0)
2	31 (34.1)	15 (31.3)	20 (28.6)
3	16 (17.6)	9 (18.8)	10 (14.3)
4	10 (11.0)	6 (12.5)	5 (7.1)
5	1 (1.1)	1 (2.1)	0

The average age of members in the elevated inattention symptom group was 10.6 (SD 3.1) years, and there was a higher proportion of children than adolescents (n=64, 70.3%). Comparatively, the elevated hyperactivity/impulsivity symptom group was about 1 year younger (mean 9.4, SD 2.8) and the elevated oppositional symptom group was about the same age (mean 10.2, SD 2.9). Indeed, 83.3% (n=40) of members with elevated hyperactivity/impulsivity symptoms were children, and there were more children than adolescents in the elevated oppositional symptom group (n=54, 77.1%). Females accounted for about half of the members in all 3 groups (elevated inattention symptoms: n=46, 50.5%; elevated hyperactivity symptoms: n=25, 52.1%; elevated oppositional symptoms: n=36, 51.4%). In all 3 groups, most members identified as White or “other.” Comprehensive demographic information for all subset groups is reported in Table 3.

**Table 3:** Member demographic information at baseline reported as a percentage or the mean (SD) for each symptom subset group.

Demographics		Inattention (n=91)	Hyperactivity (n=48)	Oppositional (n=70)
**Age (years), mean (SD)**		10.6 (3.1)	9.4 (2.8)	10.2 (2.9)
	Child (<13 years)	64 (70.3)	40 (83.3)	54 (77.1)
	Adolescent (≥13 years)	27 (29.7)	8 (16.7)	16 (22.9)
**Sex, n (%)**				
	Female	46 (50.5)	25 (52.1)	36 (51.4)
	Male	45 (49.5)	23 (47.9)	34 (48.6)
**Race/ethnicity, n (%)**				
	White	44 (48.4)	22 (45.8)	33 (47.1)
	Other	38 (41.8)	17 (35.4)	27 (38.6)
	Asian	5 (5.5)	5 (10.4)	5 (7.1)
	Hispanic/Latino	3 (3.3)	3 (6.3)	3 (4.3)
	Black/African American	1 (1.1)	1 (2.1)	1 (1.4)
	Unspecified	0	0	1 (1.4)
	American Indian or Alaska Native	0	0	0

Members in the elevated inattention, hyperactivity, and oppositional symptom groups attended between 0 and 10 total Bend Health, Inc, sessions with a coach, therapist, or psychiatrist, with the group median at 1 session (IQR 4), and all members attended at least 1 Bend Health, Inc, session with a BCC. Approximately half of the members in each group attended sessions with a psychiatrist in addition to sessions with either a coach or a therapist. Few members attended only coaching or therapy sessions (0.9% each; Table 4). Due to differences in care duration, most members in each group (65.5%-71.4%) only had a single assessment within the study time frame. For those with at least 2 assessments (n=35, 32.7%), the first and last assessments were between 0.49 and 5.52 months apart (median 1.84, IQR 2.05), and the average number of sessions for each member ranged between 1 and 4.5 times per month (mean 2.57, SD 0.85).

**Table 4:** Distribution of care practitioner type for each subset group.

Care provider	Inattention (n=91), n (%)	Hyperactivity (n=48), n (%)	Oppositional (n=70), n (%)
None (BCC^a^ only)	23 (25.3)	14 (29.2)	14 (20.0)
Coach only	1 (1.1)	1 (2.1)	0
Therapist only	0	0	1 (1.4)
Psychiatrist only	12 (13.2)	4 (8.3)	10 (14.3)
Coach and therapist	0	0	0
Coach and psychiatrist	24 (26.4)	14 (29.2)	19 (27.1)
Therapist and psychiatrist	22 (24.2)	7 (14.6)	16 (22.9)
Coach, therapist, and psychiatrist	9 (9.9)	8 (16.7)	10 (14.3)

^a^BCC: behavioral care coordinator.

### Changes in Symptom Severity

**Figure 1:**
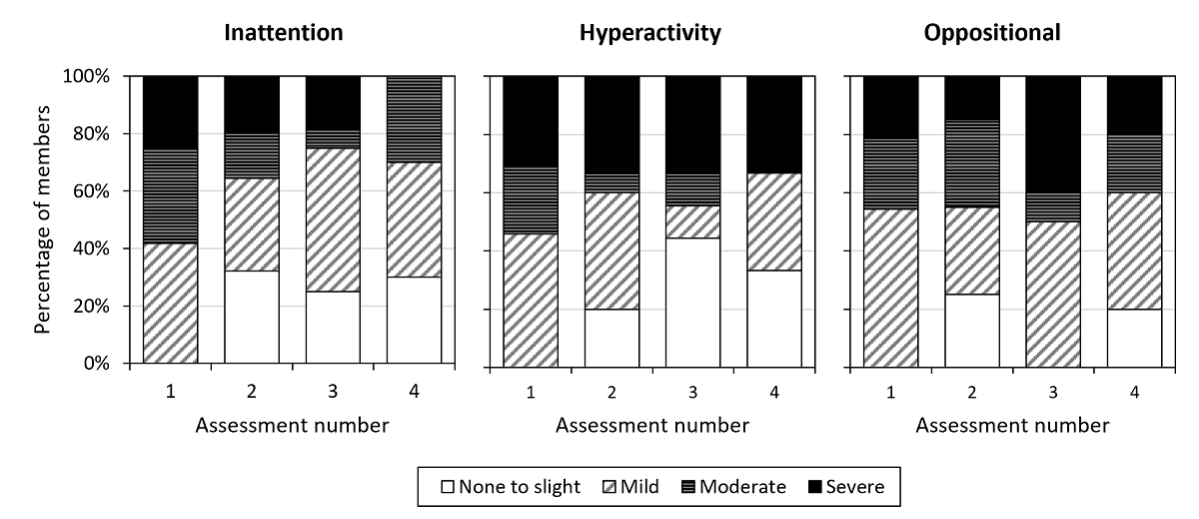
Distribution of symptom severity classification over assessments for inattention, hyperactivity, and oppositional symptoms.

At baseline, the elevated inattention symptom group’s inattention score suggested moderate symptoms (mean 19.34, SD 4.15), although the group’s symptom severity varied at baseline and across assessments (Figure 1 and Table 5). Inattention scores decreased in 71.0% (n=22) of members with at least 2 assessments, with group scores decreasing from the first assessment (moderate symptoms: mean 19.65, SD 4.31) to the last assessment (mild symptoms: mean 16.13, SD 6.17) by an average of 3.52 (SD 5.94) points (Z=–3.22, *P*=.001).

**Table 5:** Rates of SNAP-IV^a^ symptom severity categories across assessments.

Symptom severity category		Assessment 1 participants, n (%)	Assessment 2 participants, n (%)	Assessment 3 participants, n (%)	Assessment 4 participants, n (%)
**Inattention symptom severity**		91 (100)	31 (100)	16 (100)	10 (100)
	Low	0	10 (32.3)	4 (25.0)	3 (30.0)
	Mild	38 (41.8)	10 (32.3)	8 (50.0)	4 (40.0)
	Moderate	30 (33.0)	5 (16.1)	1 (6.3)	3 (30.0)
	Severe	23 (25.3)	6 (19.4)	3 (18.8)	0
**Hyperactivity symptom severity**		48 (100)	15 (100)	9 (100)	6 (100)
	None	0	3 (20.0)	4 (44.4)	2 (33.3)
	Mild	22 (45.8)	6 (40.0)	1 (11.1)	2 (33.3)
	Moderate	11 (22.9)	1 (6.7)	1 (11.1)	0
	Severe	15 (31.3)	5 (33.3)	3 (33.3)	2 (33.3)
**Oppositional symptom severity**		70 (100)	20 (100)	10 (100)	5 (100)
	None	0	5 (25.0)	0	1 (20.0)
	Mild	38 (54.3)	6 (30.0)	5 (50.0)	2 (40.0)
	Moderate	17 (24.3)	6 (30.0)	1 (10.0)	1 (20.0)
	Severe	15 (21.4)	3 (15.0)	4 (40.0)	1 (20.0)

^a^SNAP-IV: 26-item Swanson, Nolan, and Pelham.

At baseline, the elevated hyperactivity symptom group’s hyperactivity score suggested moderate symptoms (mean 19.23, SD 4.87), although the group’s symptom severity varied at baseline and across assessments (Figure 1 and Table 5). Hyperactivity scores decreased in 60.0% (n=9) of members with at least 2 assessments, with scores decreasing from the first assessment (moderate symptoms: mean 20.33, SD 4.98) to the last assessment (moderate symptoms: mean 17.27, SD 7.55) by an average of 3.07 (SD 7.24) points (Z=–1.97, *P*=.049).

At baseline, the elevated oppositional symptom group’s hyperactivity score suggested moderate symptoms (mean 14.10, SD 5.00), although the group’s symptom severity varied at baseline and across assessments (Figure 1 and Table 5). Oppositional symptom scores decreased in 60.0% (n=12) of members with at least 2 assessments from their first to the last assessment, with scores decreasing from the first assessment (mild symptoms: mean 14.05, SD 4.68) to the last assessment (mild symptoms: mean 13.35, SD 6.34) by an average of 0.70 (SD 4.87) points, although this decrease was not statistically significant (*t*_19_=–0.64, *P*=0.26).

For those with multiple assessments, 82.9% (n=29) exhibited improvements in symptom severity for at least 1 symptom type that was elevated at baseline, as indicated by a decrease in the subset score from baseline to their last assessment. Furthermore, for those in the elevated oppositional symptom group and with elevated inattention or hyperactivity symptoms at baseline, 77.8% (n=14) exhibited improvements in at least 1 of the ADHD symptom types (ie, inattention or hyperactivity).

**Figure 2:**
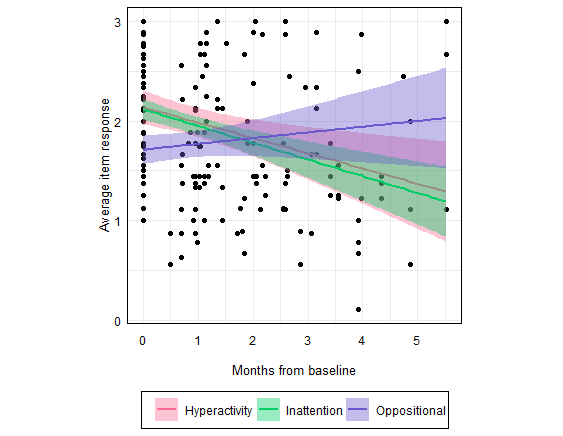
Changes in symptom severity (average item response) over time for hyperactivity, inattention, and oppositional symptoms.

The addition of each potential predictor did not improve the model fit (age: *P*=.13; sex: *P*=.26; care sessions per month: *P*=.052). Thus, the final model included the fixed effects of symptom type (inattention, hyperactivity, and oppositional) and months from baseline, their interaction, and a random effect of member on the intercept. The results from the final model are shown in Figure 2 and Table 6. Based on mixed effects ANOVA, there were statistically significant main effects of symptom type (*F*_2,221_=6.67, *P*=.002) and months from baseline (*F*_1,221_=19.75, *P*<.001) on the average item score (Figure 2). The interaction of symptom type with months from baseline was also statistically significant (*F*_2,221_=10.33, *P*<.001) such that the inattention item scores had the largest changes over time and oppositional symptoms had the smallest changes over time. With hyperactivity symptoms used as the item score reference (coefficient=0), the linear mixed effects model accounted for inattention item scores approximately equal to 0 (coefficient=0.08, *P*=.32) and significantly lower oppositional symptom scores (coefficient=–0.31, *P*<.001). The model estimated a decrease in the average item score of 0.16 points per additional month of care with Bend Health, Inc (*P*<.001) across all symptoms. However, when assessing the change in symptom severity over time by symptom type, oppositional item scores increased by an estimated 0.19 points per month (*P*<.001).

**Table 6:** Results from the primary mixed effects model of ADHD^a^ symptom severity.

		Average item response			
		Estimates (95% CI)	*P* value		
**Predictors**					
	(Intercept)	2.02 (1.87 to 2.17)	<.001^b^		
	Months from baseline	–0.16 (–0.24 to –0.08)	<.001^b^		
	Symptom type (inattention)	0.08 (–0.08 to 0.24)	.32		
	Symptom type (oppositional)	–0.31 (–0.48 to –0.14)	<.001^b^		
	Months from baseline × symptom type (inattention)	–0.02 (–0.12 to 0.08)	.68		
	Days from baseline × symptom type (oppositional)	0.19 (0.08 to 0.29)	<.001^b^		
**Random effects (** **marginal R^2^/conditional R^2^=0.111/0.462)**					
	σ^2^	0.22	N/A^c^		
	τ_00 member identification_	0.14	N/A		
	ICC^d^	0.39	N/A		
	N_member identification_	107	N/A		
	Observations, N	333	N/A		

^a^ADHD: attention-deficit/hyperactivity disorder.

^b^Significant values.

^c^N/A: not applicable.

^d^ICC: interclass correlation coefficient.

In the secondary model, including only the first 3 assessments from those with at least 3 assessments, the main effect of time (months from baseline) remained statistically significant (*F*_1,83_=14.76, *P*<.001), as did the interaction of symptom type with time (*F*_2,83_=6.79, *P*=.002). The main effect of symptom type was not statistically significant (*F*_2,83_=0.38, *P*=.68). Otherwise, the pattern of results remained consistent with the primary analysis (Multimedia Appendix 1, Supplementary Table 1).

## Discussion

### Principal Findings

The purpose of this study was to use member (ie, child and adolescent) data from Bend Health, Inc, a collaborative care DMHI that uses a whole-family approach to address child and adolescent mental health concerns, to (1) determine the effects of a collaborative care DMHI on inattention, hyperactivity, and oppositional symptoms in children and adolescents and (2) assess whether the effects of a collaborative care DMHI vary across ADHD subtypes and demographic factors. To the best of our knowledge, this is the first study to test whether the symptoms of ADHD and ODD decrease significantly among children and adolescents involved in a collaborative care DMHI. We found that most members exhibited improvements in inattention, hyperactivity, or oppositional symptom severity from their first to their final assessment, with significant decreases in inattention and hyperactivity scores. Although oppositional scores increased slightly over time, symptom severity significantly decreased over time regardless of symptom type. Finally, the frequency of sessions with a Bend Health, Inc, care provider was not a useful predictor of symptom severity or change over time.

We found that the symptoms of ADHD and ODD improved for most of those engaged with Bend Health, Inc, from baseline to their last assessment. Specifically, inattention symptoms improved in 71.0% of members, hyperactivity symptoms improved in 60.0% of members, and oppositional symptoms improved in 60.0% of members. Further, scores decreased significantly from baseline for inattention and hyperactivity symptoms. These results offer promising early evidence that participation in collaborative care DMHIs is linked to reduced symptoms of ADHD. This work adds to our previous finding that participation in a collaborative care DHMI is linked to significant reductions in internalizing symptom severity in children and adolescents [31]. Previous studies have demonstrated the efficacy of nonpharmacological approaches in treating ADHD, including behavioral care (eg, coaching and therapy) [10] and digital games (eg, *EndeavourRX*) [33,34,36]. Although ADHD is 1 of the most common diagnoses in children and adolescents referred to digitally delivered care, the use of collaborative care DMHIs to treat ADHD remains uncommon. Extant studies of DMHIs offer mixed results: for example, there is evidence that teletherapy may improve oppositional symptoms but not inattention and hyperactivity symptoms [37]. Others have cited individual case studies of improvement in ADHD symptoms over the duration of care with a DMHI [38]. Clinicians consistently recommend including behavioral care as a core component of ADHD treatment [2]. However, approximately 1 in 4 children and adolescents with ADHD are not receiving any form of treatment [7], largely due to a lack of geographical or financial accessibility [25,44]. Moreover, among those being treated for ADHD, an estimated 30% are treated with medication alone [1]. Here, we showed that DMHIs may bridge the need for affordable, accessible, and high-quality behavioral care for those with ADHD and oppositional symptoms.

We found that the length of involvement has a positive association with symptom change such that greater durations of care with Bend Health, Inc, are related to greater improvements in symptom severity. Our results are bolstered by others’ findings that suggest nonpharmacological interventions, including therapy, are effective treatments for inattention and hyperactivity symptoms [9]. However, we found that those with oppositional symptoms exhibited slight increases in symptom severity over time. Notably, 81.4% of the members in the elevated oppositional symptoms group also had elevated symptoms of inattention, hyperactivity, or both, and 77.8% of those exhibited improvements on at least 1 other symptom type that was elevated at baseline. Thus, despite resistance of oppositional symptoms to improvement in this study, our results demonstrate that collaborative care with a DMHI is associated with positive changes in the symptoms of ADHD and ODD overall. A large body of literature suggests that antisocial behavioral disorders, such as ODD, are typically less plastic throughout the life span and more difficult to address with interventions [45]. As such, the suggested treatments for oppositional and defiant behaviors in children typically include multisystemic interventions, including parent training and community-based approaches [5,46]. Within the context of this knowledge, the trend of oppositional symptoms observed in this study may stem from the resistance of ODD to interventions *or* the limited number of methodological predictors (eg, length of care and sessions per month) considered here. Because Bend Health, Inc, follows a whole-family approach, and parent-training techniques are delivered to caregivers in sessions and through online resources, future Bend Health studies should measure and test the associations between these specific behavior interventions and symptom progression.

Finally, we found that the session frequency with Bend Health, Inc, does not predict symptom outcomes. Our previous study of Bend Health reported that more sessions per month are related to lower levels of depressive and anxiety symptom severity [31]. Notably, the addition of session frequency as a predictor in the main analysis trended toward statistically improving model fit and our data set may have been too small to investigate nuances in various factors. Indeed, although our results provide compelling support for the use of collaborative care DHMIs in treating ADHD symptoms in children and adolescents, future studies (eg, with larger sample sizes) should further investigate the potential associations between ADHD symptom severity and demographic and methodological factors.

### Limitations and Future Directions

Although our findings offer promising preliminary evidence for the merits of collaborative care DHMIs in treating children and adolescents with ADHD symptoms, this study is limited by several factors. First, this was a retrospective study and did not include a control group, such as individuals receiving in-person care or those receiving nonactive treatment (eg, sham control). As such, this study cannot make any causal inferences nor assess whether collaborative care DHMIs may deliver similar or different results than standard or alternative behavioral care. Indeed, future studies would benefit from the inclusion of nonactive controls to further parse apart the effectiveness of Bend Health, Inc, and other collaborative care DHMIs in addressing the symptoms of ADHD and ODD. Similarly, the data used for this study are limited in that members participated in Bend Health, Inc, for a relatively moderate duration of care (up to 221 days), so we could not assess the long-term or lasting effects of behavioral care with a collaborative care DHMI.

We were unable to thoroughly investigate the role of several potential covariates, including race/ethnicity, gender conforming status, and type of care because of this study’s small sample size. Further, due to a limitation in the self-report race/ethnicity measure used in this study, a third of the present sample reported their race/ethnicity as “other” instead of indicating a particular ethnic minority. Given that ethnic minorities often experience greater difficulties receiving diagnoses and obtaining care for mental and behavioral health problems, future studies should investigate racial/ethnic differences beyond White versus non-White status [22,40]. Moreover, few gender nonconforming youth were represented in this sample. This is particularly important for ADHD, as higher levels of gender nonconformity have been reported in children and adolescents with ADHD [47]. In this study, we were unable to investigate whether differences in the type of care (eg, coaching, therapy, psychiatry, or a combination of types of care) was linked to differences in outcome. Although therapy and psychiatry often go hand in hand in the collaborative care model, which has emerged as a paragon of mental and behavioral health care, the nascency of behavioral health coaching means that little is known regarding its effectiveness alongside other types of care. Future research among Bend Health, Inc, members should investigate these care-based differences.

Lastly, we did not assess the potential role of pharmacological treatment in ADHD-related outcomes of children and adolescents. Given that medication in conjunction with behavioral therapy is currently the recommended treatment for ADHD [2], we would expect that medication plays an important role in changes in symptom severity over time among those participating in behavioral health care. However, only 2%-3% of Bend Health, Inc, members are prescribed medication during their treatment, suggesting that inclusion of medication information would not significantly change the results of our analyses. Further, information regarding existing medical treatment is gathered through caregiver reports upon enrollment with Bend Health, Inc, and caregiver reported data on medication type, dose, and frequency may be inaccurate. Finally, the potential associations between outcomes and types of treatment were beyond the scope of this project. Future studies, with a larger sample of individuals taking medication, should investigate whether behavioral treatment versus behavioral treatment *and* pharmacological treatment are associated with different outcomes.

### Conclusion

This study provides preliminary evidence that collaborative care DMHIs, such as Bend Health, Inc, may aid in reducing the symptoms of ADHD among children and adolescents, particularly symptoms of inattention. Future studies should include more robust research designs, such as inclusion of a randomized or waitlist control group. Moreover, future research should evaluate the following: (1) the role of methodological factors and demographics in ADHD symptom changes over time among those participating in a collaborative care DMHI; (2) the potential added benefits of care via a collaborative care DMHI compared to alternative interventions (eg, medication and school support); (3) changes in stress, well-being, and positive parenting outcomes among parents whose children are participating in a collaborative care DMHI; and (4) self-reported ADHD symptom severity and acceptance of care via a collaborative care DMHI.

## Appendix

Multimedia Appendix 1 Results from the secondary mixed effects model of ADHD symptom severity, including only the first 3 assessments from those with at least 3 total assessments.

## Abbreviations

ADHD: attention-deficit/hyperactivity disorder
BCC: behavioral care coordinator
CBT: cognitive behavioral therapy
DMHI: digital mental health intervention
ODD: oppositional defiance disorder
PCP: primary care provider
SNAP-IV: 26-item Swanson, Nolan, and Pelham

## Data Availability

The data sets analyzed during this study are not publicly available, as this would violate Bend Health, Inc’s, privacy policy. However, aggregated and anonymized data that are not associated with individual users and do not include personal information are available from the corresponding author upon reasonable request.
