# Supplementary material for: Using Digital Measurement–Based Care to Address Symptoms of Inattention, Hyperactivity, and Opposition in Youth: Retrospective Analysis of Bend Health
Source: JMIR Form Res. 2023 Apr 26;7:e46578. doi: 10.2196/46578 (PMC10173032; doi:10.2196/46578)
Supplement: Multimedia Appendix 1 [file formative_v7i1e46578_app1.docx]

**Supplementary Table** **1.** Results from the secondary mixed effects model of ADHD symptom severity, including only the first 3 assessments from those with at least 3 total assessments.

|  | Average item response | | |
| --- | --- | --- | --- |
| *Predictors* | *Estimates* | *CI* | *P* |
| (Intercept) | 2.21 | 1.81 – 2.53 | **<.001** |
| Months from baseline | -0.24 | -0.37 – -0.11 | **.001** |
| Symptom type [Inattention] | 0.01 | -0.33 – 0.36 | .94 |
| Symptom type [Oppositional] | -0.31 | -0.70 – -0.07 | .11 |
| Months from baseline x Symptom type [Inattention] | 0.00 | -0.17 – 0.18 | .99 |
| Days from baseline x Symptom type [Oppositional] | 0.28 | 0.10 – 0.46 | **.003** |
| **Random effects** |  |  |  |
| σ^2^ | 0.22 |  |  |
| τ_00 Member ID_ | 0.22 |  |  |
| ICC | 0.50 |  |  |
| N _Member ID_ | 17 |  |  |
| Observations | 105 |  |  |
| Marginal R^2^ / Conditional R^2^ | 0.132 / 0.568 | | |
